# Retroperitoneal paraganglioma manifesting as paralytic ileus: a case report

**DOI:** 10.1186/1752-1947-6-158

**Published:** 2012-06-20

**Authors:** Wei-Chen Lin, Horng-Yuan Wang, Chen-Wang Chang, Jiun-Lu Lin, Chung-Hsin Tsai

**Affiliations:** 1 Division of Gastroenterology, Department of Internal Medicine, Mackay Memorial Hospital, Taipei, Taiwan; 2 Division of Endocrinology and Metabolism, Department of Internal Medicine, Mackay Memorial Hospital, Taipei, Taiwan; 3 Department of General Surgery, Mackay Memorial Hospital, Taipei, Taiwan; 4 Mackay Medicine, Nursing and Management College, Taipei, Taiwan

## Abstract

**Introduction:**

Retroperitoneal neoplasms are rare and easily misdiagnosed. These tumors are often discovered incidentally during imaging studies performed for other reasons. Paragangliomas are tumors that arise from extra-adrenal medullary neural crest derivatives. They are usually located in the head and neck but can be found in various body sites, including the chest cavity, abdomen, pelvis and bladder. We report the case of a patient who had a retroperitoneal paraganglioma manifested as paralytic ileus, which is an unusual presentation of a paraganglioma.

**Case presentation:**

A 63-year-old Taiwanese woman was admitted to the emergency department of our hospital with progressive abdominal fullness for two days. Her medical history included medically controlled hypertension for 10 years and type 2 diabetes mellitus. Plain abdominal radiography showed a solitary loop of the air-filled dilated small bowel. Abdominal computed tomography did not show a mechanical obstruction; however, a retroperitoneal mass was incidentally detected. Histological analysis of the mass led to a diagnosis of a paraganglioma.

**Conclusions:**

In cases of patients with hypertension presenting with an intestinal pseudo-obstruction, a paraganglioma may be considered as a possible differential diagnosis of retroperitoneal tumors to avoid risky therapeutic procedures or medication that may produce severe adverse effects.

## Introduction

Retroperitoneal neoplasms are rare tumors and present several therapeutic challenges because of their rarity and relatively late presentation, with soft tissue sarcoma being the most common type. Paragangliomas are one of these retroperitoneal tumors that mainly affect adults who are in the fourth or fifth decade of life, and they have no sex predilection. Approximately 10% of patients with a paraganglioma have a significant family history and one-third of cases are associated with germ line mutations in at least nine genes (*NF1RETSDHASDHBSDHCSDHDTMEM127MAX* and *VHL*) [1].

Most paragangliomas are located in the head and neck and less than 5% of these tumors are endocrinologically active [2,3]. Except for the paragangliomas that originate from the organ of Zuckerkandl and produce various catecholamines, functional paragangliomas in various body sites secrete only noradrenaline owing to a lack of phenylethanolamine *N*-methyltransferase in those areas. Most paragangliomas with catecholamine hypersecretion are localized to the abdomen and pelvis [2]. Patients with secretory tumors experience paroxysmal episodic hypertension, as well as the typical triad of symptoms associated with pheochromocytomas, for example, palpitations, headache and sweating. Nonfunctional paragangliomas most commonly manifest as abdominal pain or a mass [4]. In this report, we present a rare case of a retroperitoneal paraganglioma that manifested as paralytic ileus.

## Case presentation

A 63-year-old Taiwanese woman was admitted to our emergency department because she experienced progressive abdominal fullness, nausea and intermittent dull epigastric pain for two days. Relieving or aggravating factors were not present. Her past medical history included well controlled hypertension for 10 years; type 2 diabetes mellitus; and acute appendicitis that occurred after an operation performed 10 years ago. Her blood pressure was 177/100 mmHg and heart rate was 86 beats/minute on arrival at our hospital. Her abdomen was mildly distended and tympanic with periumbilical tenderness on palpation. Bowel sounds were hypoactive and the remaining examination yielded normal results. Laboratory investigations showed leukocytosis (white blood cell count: 16,500 cells/uL) and unremarkable amylase and lipase values. Carcinoembryonic antigen and cancer antigen 19–9 levels were within normal limits.

**Figure 1:**
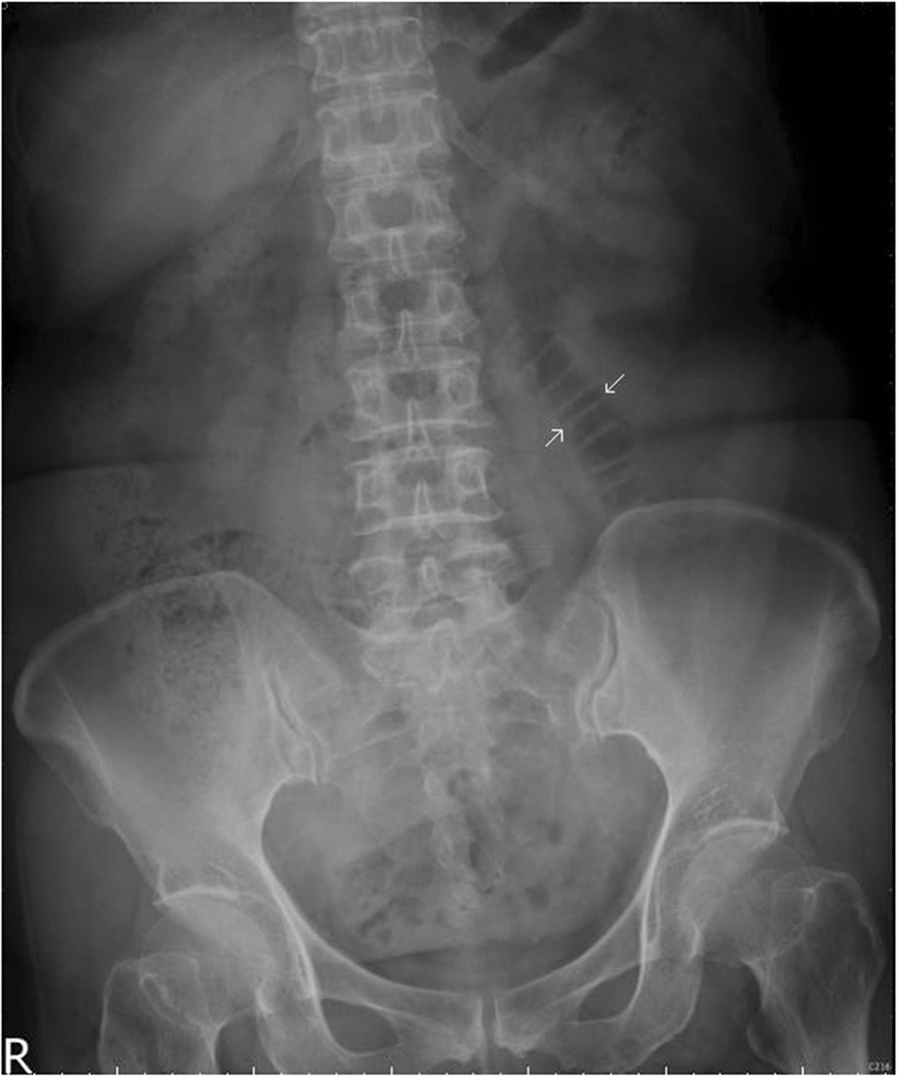
**Plain film findings.** A solitary dilated loop of small bowel was in the left upper quadrant (arrow).

**Figure 2:**
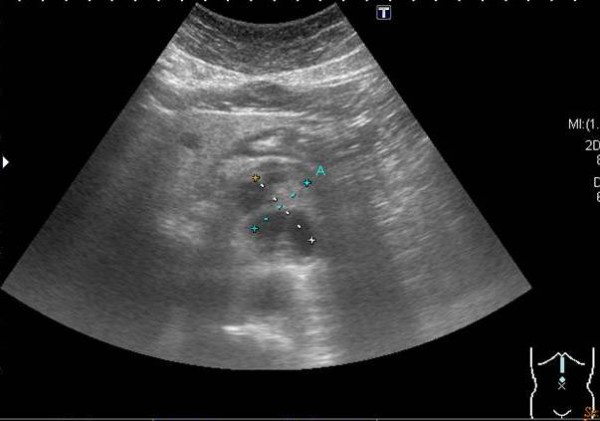
**Ultrasonography findings.** A mix-echoic lesion was near the pancreas.

**Figure 3:**
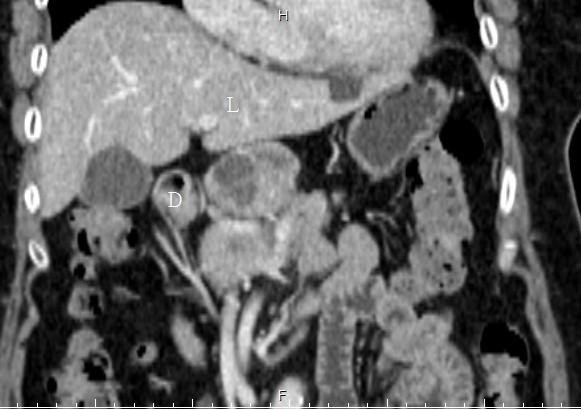
**Computed tomography findings.** A mass was between the duodenum (**D**) and liver (**L**).

A plain radiograph showed a coiled-spring appearance of her small bowel in the left upper quadrant (Figure 1). Ultrasonography showed a mixed-echoic lesion (diameter approximately 4 cm) near the pancreatic body (Figure 2). A computed tomography (CT) scan of her abdomen incidentally showed a hypervascular retroperitoneal mass that had central areas of low attenuation, caused displacement of the anterior aspect of the pancreatic body, and was in proximity to the caudate lobe of her liver (Figure 3).

**Figure 4:**
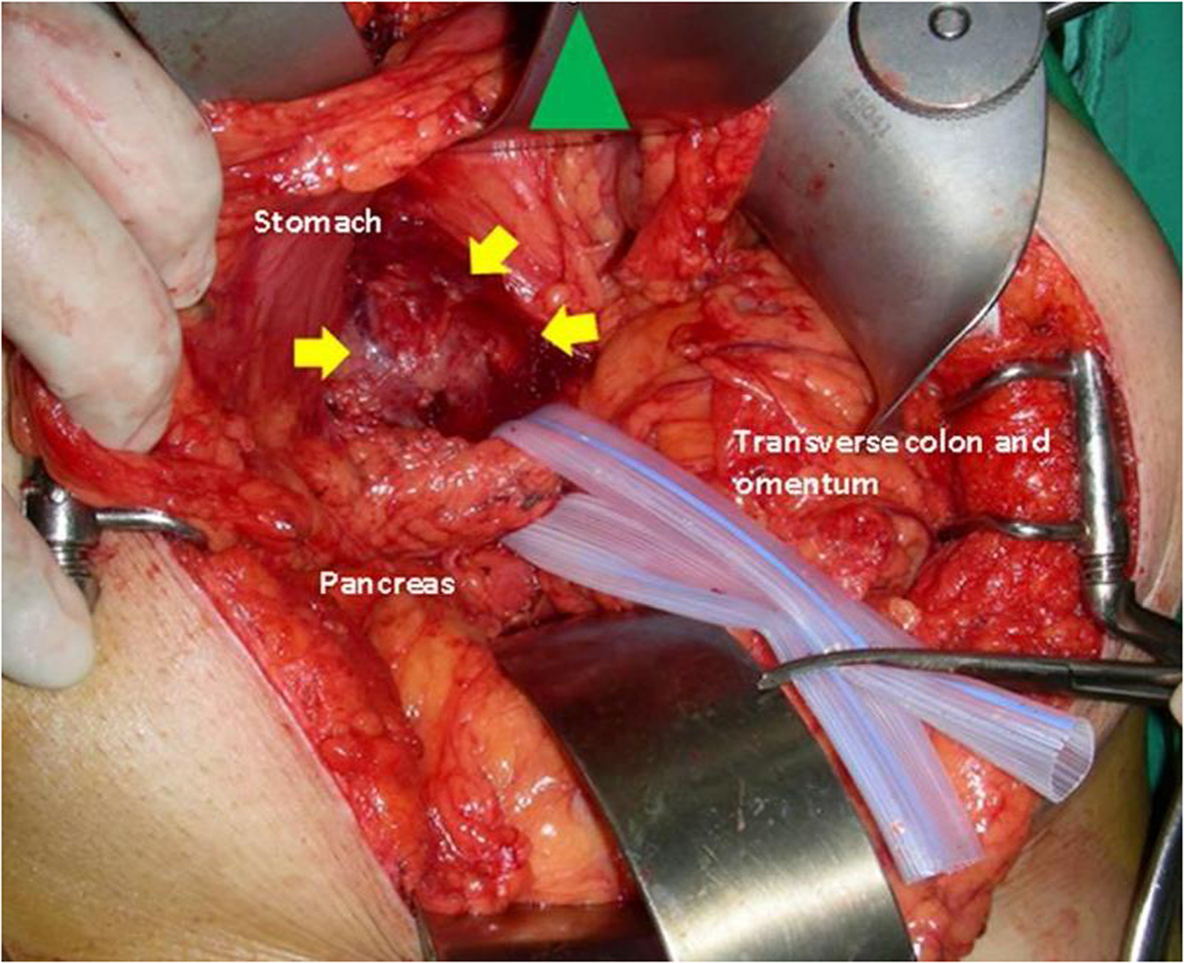
**Intra-operative photograph.** A well-demarcated mass was in the peritoneal cavity.

**Figure 5:**
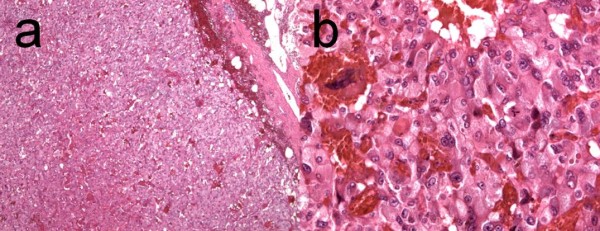
**Pathology findings. (a)** Central necrosis and hemorrhage was noted in this capsulated tumor (hematoxylin and eosin × 5). **(b)** The tumor cells contained abundant oncocytic cytoplasm and pleomorphic nuclei and were arranged in sheets, large nests and organoid patterns (hematoxylin and eosin × 40).

Our patient received surgical treatment after full fasting and adequate hydration. At the time of surgery, a well-demarcated, hypervascular, fragile mass (approximately 6.5 cm × 6.5 cm × 4 cm) was observed in her retroperitoneal area and was subsequently excised (Figure 4). Pathological analysis of the mass showed that it was a capsulated tumor with central necrosis and hemorrhage; the tumor cells were arranged in sheets, large nests and organoid patterns, and contained abundant oncocytic cytoplasm and pleomorphic nuclei (Figure 5). The positive results of immunohistochemical staining for chromogranin A, cluster of differentiation 56 and vimentin were consistent with the presentation of a paraganglioma. Urinary fractionated catecholamine levels and fractionated plasma metanephrine levels were within normal limits and our patient recovered uneventfully after surgery.

## Discussion

Pheochromocytomas and extra-adrenal paragangliomas are very rare tumors that arise from the neural crest tissue. These tumors are functional in more than half of such cases, and patients commonly present with symptoms, such as palpitation, headache, sweating and hypertension, that are related to excess catecholamine secretion [2]. The typical gastrointestinal manifestations include nausea, vomiting, constipation and abdominal pain. Standard internal medicine textbooks do not include paragangliomas in the differential diagnosis of ileus; however, in several case reports, patients with pheochromocytomas have been found to present with ileus. Our patient received triple-antihypertensive drug combination therapy, that is, therapy with calcium channel blockers, angiotensin II receptor blockers and beta-blockers, for hypertension. She presented with paralytic ileus, and there was no hypertensive crisis or palpitation during admission. The ileus improved after full fasting, adequate hydration and tumor resection.

Intestinal pseudo-obstruction is a rare complication of these tumors. The underlying mechanism is catecholamine-induced relaxation of the intestinal smooth muscles, which is mainly effected by the activation of alpha-adrenergic receptors, thereby inhibiting the release of acetylcholine from the postganglionic nerve terminals. Subsequently, the net effect is the depression of peristalsis and constriction of the sphincters, thereby inducing ileus. The other underlying mechanism involves catecholamine-induced vasoconstriction of the mesenteric vessels. The results of a literature review showed that ileus is related to large tumors and high catecholamine levels [5]. In several case reports of patients with pheochromocytomas that manifested as ileus [5-8], the severity of the hypertensive crisis was less in the patients with a history of hypertension than in those without a history of hypertension. In these studies, the ileus improved after resection of the tumor or administration of an alpha-adrenergic antagonist. Other than serum catecholamines, opioid-like peptides that are secreted by pheochromocytomas [9] may play a role in the development of ileus.

A prokinetic agent like metoclopramide is often used in cases of decreased gastrointestinal myoelectric activity and motility. However, metoclopramide worsens the symptoms of pseudo-obstruction owing to its catecholamine-stimulating effect, subsequently inducing a hypertension crisis. Therefore, metoclopramide is contraindicated in patients with a suspected pheochromocytoma or paraganglioma. The initial management of paralytic ileus caused by a paraganglioma involves providing the patient with adequate alpha-blockers and hydration [7].

Several radiologic imaging and nuclear imaging techniques are currently available for evaluating extra-adrenal paragangliomas. On CT images, a retroperitoneal paraganglioma appears as a hypervascular mass. A high rate of preoperative CT misdiagnosis (17 out of 19, 89%) was reported in a 2010 study [10] involving the retrospective analysis of 19 patients who were pathologically diagnosed with retroperitoneal paragangliomas. Magnetic resonance imaging (MRI) is more sensitive than CT in detecting extra-adrenal tumors. Metaiodobenzylguanidine (MIBG) scintigraphy is used to differentiate between functioning and nonfunctioning paragangliomas; however, its sensitivity is quite low when compared with that of CT and MRI [3,11]. Somatostatin receptor scintigraphy is found to be more sensitive than MIBG scintigraphy in patients with highly suspected head and neck paragangliomas [11].

Besides the use of imaging studies for diagnosing paragangliomas, assessment of the plasma normetanephrine level or of the metanephrine and catecholamine levels via 24-hour urine collection can help physicians make the actual diagnosis. In our patient’s case, diagnosing the paraganglioma before surgery was challenging; furthermore, we did not analyze the relevant serum marker. Complete surgical excision is the treatment of choice for extra-adrenal paragangliomas as well as for recurrent or metastatic neoplasms. Urinary fractionated catecholamine and metanephrine levels or plasma-fractionated metanephrine levels should be measured after surgery to evaluate residual tumor or occult metastasis. Follow-up imaging is necessary in cases of patients with elevated metanephrine and catecholamine levels or with nonfunctional original tumors [2]. Close, lifelong follow-up is necessary and critical in the aforementioned conditions [12].

## Conclusions

Preoperative diagnosis of retroperitoneal neoplasms is difficult. Paragangliomas are very rare entities with a limited number of cases reported, and paralytic ileus is a rare manifestation of a paraganglioma. In cases of patients with hypertension presenting with an intestinal pseudo-obstruction, a paraganglioma may be considered as a possible differential diagnosis of a retroperitoneal tumor. Prokinetic agents could worsen the ileus and cause malignant hypertension. Surgical resection is necessary for histological assessment and remains the mainstay of paraganglioma treatment.

## Consent

Written informed consent was obtained from the patient for publication of this case report and any accompanying images. A copy of the written consent is available for review by the Editor-in-Chief of this journal.

## Competing interests

The authors declare that they have no competing interests.

## Authors’ contributions

CC and CT diagnosed, investigated, followed-up and managed the patient, and HW determined the medical significance. WL wrote the manuscript. CC and JL revised the manuscript. HW provided important suggestions regarding medical content. All authors read and approved the final manuscript.
